# Pilot study of personalized sleep-coaching messages to promote healthy sleeping behaviors

**DOI:** 10.3389/frsle.2022.1071822

**Published:** 2023-01-09

**Authors:** Logan D. Schneider, Andrew Barakat, Zainab Ali, Christian Concepcion, James A. Taylor, Allen Jiang

**Affiliations:** ^1^Alphabet, Inc, Mountain View, CA, United States; ^2^Stanford Sleep Center, Redwood City, CA, United States; ^3^Stanford/VA Alzheimer's Research Center, Palo Alto, CA, United States; ^4^Pacific Bioscience, Menlo Park, CA, United States; ^5^School of Medicine, University of Washington, Seattle, WA, United States

**Keywords:** coaching, behavior change, sleep, wellness, health

## Abstract

**Objectives:**

Assess a program of sleep schedule recommendations and behavior change “nudges” algorithmically selected using passively collected, longitudinal sleep data. Improvements were primarily measured by sleep schedule adherence and changes in sleep health (quantified by the RU_SATED framework).

**Methods:**

This study used a convenience sample of self-screening volunteers, responding to recruitment emails. Sleep data was gathered with a commercial under-mattress sensor through three phases: baseline passive data collection-2 weeks; intervention-4 weeks; maintenance monitoring passive data collection-8 weeks. The intervention included sleep schedule recommendations and SMS “nudges,” based on rules and recommendations derived from the extant literature. A daily sleep-health score (based on RU_SATED) was derived from passively collected sleep data and daily self-reports of alertness and sleep quality.

**Results:**

Twenty-six participants (34.92 ± 10.08-years-old; 20M:6F) had adequate data for analysis. The main findings were: (1) Adherence—defined as a wake time (WT) within 30 min of the recommendation—rates did not differ significantly between the three study phases. However, there was a general decline in adherence over the course of the study, with adherence rates dropping by about 1.5%/week in a pattern of progressive delay of WTs. (2) Linear mixed models (LMMs) of individual sleep metrics did not demonstrate a significant change over the course of the intervention, possibly due to widely varying, yet relatively healthy, sleep patterns at baseline. (3) Comparatively, the composite, sleep-health (RU_SATED) score demonstrated general improvement over the intervention period, in association with higher rates of WT adherence.

**Conclusions:**

While, in general, adherence to a sleep schedule and individual dimensions of sleep health did not demonstrate meaningful improvements during the intervention phase, those individuals that were more consistently meeting the wake-time schedule recommendation had associated improvements in their overall sleep-health. As such, this pilot study demonstrates the feasibility and potential efficacy among more adherent individuals of implementing a sleep wellness coaching framework using passively collected sleep data and a rule-based coaching infrastructure.

## Introduction

Inadequate sleep duration has been declared a public health epidemic in the United States, with over $\frac{1}{3}$ of Americans not getting the necessary amount of sleep on a nightly basis (CDC, 2019). On a global scale, a similar proportion of people aren't getting sufficient amounts of sleep every night (Chattu et al., 2018). There are various reasons that may explain this concerning trend, some of which are behavioral [e.g., bedtime procrastination due to technology usage (Chung et al., 2020)]. In order to tackle this pervasive problem, numerous organizations have attempted to promote public awareness through publicizing guidelines on sleep duration recommendations for adult and pediatric populations (Hirshkowitz et al., 2015; Watson et al., 2015; Paruthi et al., 2016; Tremblay et al., 2016; World Health Organization, 2019; Ross et al., 2020).

However, sleep duration alone seems an inadequate measure of sleep health, given the growing body of evidence supporting various attributes of daily sleep-wake patterns that meaningfully associate with functional and health outcomes (Buysse, 2014). Toward this end, sleep health frameworks, like the RU_SATED paradigm conceptualized and validated by Buysse and colleagues (Brindle et al., 2019; Wallace et al., 2021), can help define healthy sleep akin to other wellness goals, such as the American Heart Association's (AHA) weekly exercise recommendations (Piercy Katrina and Troiano Richard, 2018). The RU_SATED framework captures 6 dimensions of sleep health: **R**o**U**tine/regularity (consistency of the sleep schedule from day to day), **S**leep quality (perceived and/or objective), **A**lertness (ability to remain awake), **T**iming (when in the 24 hours the sleep period occurs), **E**fficiency (the match between sleep opportunity and sleep duration), and **D**uration (adequacy of sleep quantity). In this vein, a focus can be placed on wellness behaviors throughout the day and night that promote healthy sleep. But, given the complexity of interactions between daily activities and sleep, as well as needs for individuals varying widely based on various life factors, applying the best scientific evidence to each person's unique circumstances and sleep-health profile poses a significant challenge. This is underscored by growth of consumer investment in sleep-health-promoting products, which are focused on addressing issues that generally don't fall under the purview of clinical sleep medicine (Khosla et al., 2018).

Along these lines, various consumer-facing sleep technologies have sought to help individuals understand their sleep, often building upon some form of sleep-tracking technology. Many of these efforts provide a more retrospective view of sleep data that has resulted in some dissatisfaction with the lack of guidance (Chen, 2019) on how to achieve better sleep or, in occasional circumstances, data-driven sleep impairments, which has been dubbed “orthosomnia (Baron et al., 2017).” Moreover, behavior change is hard to initiate and maintain. Meta-analyses have demonstrated that adherence rates for various health interventions tend to range from 50 to 80%, with the ability to sustain healthy behaviors in the long-term failing in 30–60% of cases (Middleton et al., 2013). Even in instances where individuals are seeking care for a specific sleep disorder—insomnia—digital clinical interventions (i.e., cognitive behavioral therapy for insomnia [CBTi]) tend to garner only about 52% adherence, on average (Horsch et al., 2015).

Recognizing the prevalence of both sleep complaints and unrecognized sleep issues that may be detracting from optimal sleep health (Grandner, 2019), we sought to perform a pilot study to explore whether sleep coaching messages based on longitudinally monitored, objective, sleep-tracking data could help volunteers make changes to their daily behaviors in order to improve their overall sleep health. We hypothesized that algorithmically derived sleep schedule recommendations and “personalized” sleep coaching messages would (1) result in adherence rates comparable to other health and wellness interventions and (2) improve objective measures of sleep in a population of generally healthy volunteers free of clinically diagnosed sleep disorders.

## Methods

This study was approved by an institutional review board (IRB), protocol # GH-SBC-001. All participants provided written, informed consent prior to participation in the study.

### Participants

Research participants were recruited as a convenience sample. Volunteers were sought from within Google LLC *via* emails sent to relevant full-time employee listservs. There was no incentive offered for participation and no punitive consequences for lack of participation or completion of the study. Interested volunteers were asked to complete a screening questionnaire to determine eligibility for study enrollment, based on the inclusion/exclusion criteria (Table 1). Because the sleep schedule recommendation and coaching messages were based on sleep-related behaviors, individuals with sleep schedule irregularity—defined as a >1 h difference in bedtime and/or wake time, at least once a week, on a regular basis—were preferentially recruited.

**Table 1:** Inclusion and exclusion criteria.

**Inclusion criteria**
1. Google full-time employee 2. Sleep schedule irregularity 3. Age >18 4. Speak and read English 5. Own a smartphone with texting capability
**Exclusion criteria**
1. Have more than 2 sleepers in the bed 2. Share bed with children or pets 3. Have an expected use of own bed of <80% of the time 4. Sleeps on waterbeds or air mattresses 5. History of the following medical conditions: significant cardiopulmonary disease, significant neurologic disease, significant mental illness, obstructive sleep apnea, specific sleep disorder as diagnosed by a physician 6. Current pregnancy or planned pregnancy during the course of the study 7. Unwillingness or inability to comply to actions suggested by the study (for any reason)

### Study protocol

**Figure 1:**
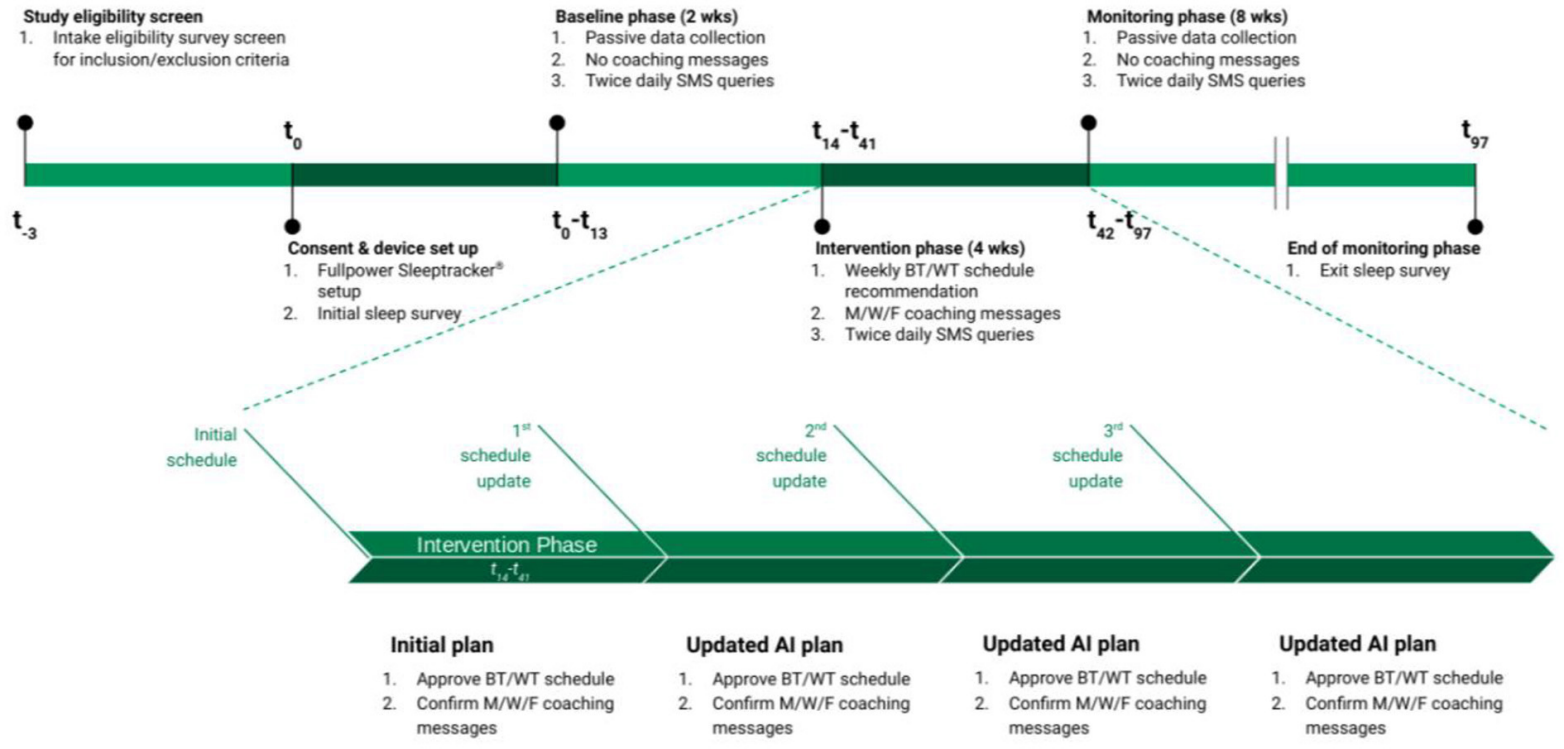
Study design. Following consent, participants had their sleep passively monitored by the Fullpower Sleeptracker^®^ for 2 weeks (Baseline phase) along with twice daily SMS queries. On the 14th day of the study, participants entered the Intervention phase, a 4-week phase in which automatically generated sleep schedule recommendations—bedtime (BT) and wake time (WT)—and a sleep insight topic and messages were confirmed by team consensus after review of the collected sleep data. After completion of the Intervention phase, the participants continued with passive sleep monitoring and twice daily SMS queries for another 8 weeks (Monitoring phase). AI, active intervention; M/W/F, Monday/Wednesday/Friday; SMS, short messaging system.

Individuals participated in the study for a total of 14 weeks (Figure 1). After providing informed consent, participants set up the Fullpower Sleeptracker^®^ under-mattress sensor (“the sensor”) to start passively monitoring their sleep patterns (Ding et al., 2022). This data was abstracted to a secure spreadsheet for analysis on a daily basis. Throughout the study, participants did not have access to their data or the features of the Fullpower Sleeptracker^®^ app. Participants also received twice daily SMS messages to survey their perceived sleep quality—Overall, how was the quality of your sleep last night? “1” (very poor) to “5” (very good)—and daytime alertness levels—How alert were you today? “A” (not at all alert) to “E” (most alert); the quality SMS survey was sent within an hour of usual wake time, in order to get an accurate reflection on the previous night's sleep without the bias of sleep inertia, and the alertness SMS survey was sent about 6 h before usual bedtime, in order to follow the usual midday, circadian dip in energy levels. This data was also added to the secure spreadsheet for analysis and team decision-making.

### Entry and exit surveys

At the beginning of the study, and again at the end of the 14 weeks, participants completed a brief survey about their age, self-identified sex, general sleep habits (e.g., usual bedtime, average sleep duration, etc.), perceived sleep issues, and sleep goals. The exit survey focused on feedback regarding the coaching experience (e.g., frequency of SMS, relevance of content, etc.). Neither survey was used in the Intervention phase of the study, but the data were gathered for qualitative feedback on the coaching experience and, in the case of age and sex, for model adjustment in quantitative analyses.

### Baseline phase

The first 2 weeks of the study included only passive sleep pattern monitoring and the twice daily SMS text messages.

### Intervention phase

Following 2 weeks of sleep pattern and quality/alertness data collection, participants entered the 4-week intervention phase. In addition to the ongoing sleep monitoring and twice-daily SMS surveys, individuals received a sleep schedule recommendation (recommended bedtime and wake time) and thrice-weekly sleep coaching messages. The sleep schedule recommendations were automatically calculated from the collected data from the previous 14 days. After the research team (blinded to participant identity) reviewed the sleep data and approved the sleep schedule recommendation, the recommended bedtime and wake time were shared with the participant *via* SMS and entered into a Google Calendar, which were associated only to the participant's de-identified study ID. The research team also reviewed the relevant coaching messages that were identified by a series of rules that automatically analyzed the collected sleep data (see “Coaching message selection” subsection). Once the coaching messages were confirmed to be appropriate by consensus opinion, they were sent *via* SMS, using the same platform as the twice-daily survey questions. In order to standardize and simplify the study process, the research team met on Monday, Wednesday, and Friday in order to review the participant data and schedule/coaching message recommendations.

### Monitoring phase

Following the 4-week Intervention phase, participants were again passively monitored with the under-mattress sensor and asked to respond to twice-daily SMS survey questions about their sleep quality and daytime alertness levels.

### Sleep schedule recommendation

Each week, the preceding 14 days of data were used to generate a sleep schedule recommendation. In order to focus on regularity of the sleep schedule, bedtime and wake time recommendations were generated from usual bedtimes and wake times, accounting for the relationship between the usual sleep duration and nightly sleep opportunity. Prior to providing recommendations, all schedule recommendations were reviewed by the research team to ensure adequate sleep opportunity, based on the participant's historical data. If the recommended schedule was deemed inappropriately short or long, the team would override the recommendation, instead suggesting a 7- or 9-h sleep period, respectively, in accordance with generally accepted healthy sleep guidelines for this demographic (Hirshkowitz et al., 2015; Watson et al., 2015).

### Coaching message selection

As part of the study protocol, sleep coaching messages were generated that helped identify the area of potential sleep improvement, provide education on basic principles of sleep-wake physiology, and suggest an action that could be undertaken to make the intended improvement. This content was developed based on the extant body of peer-reviewed scientific literature and covered various concepts (e.g., the homeostatic sleep drive, the circadian system, etc.). Sleep improvement areas were identified, based on a number of evidence-based rules derived from peer-reviewed publications, professional society guidelines (e.g., American Academy of Sleep Medicine, National Sleep Foundation, etc.), and general health and wellness principles that are discussed in public outreach messages from reputable organizations (e.g., MayoClinic.org). The sleep improvement areas focused on multiple aspects of healthy sleep, generally structured around the RU_SATED paradigm (Buysse, 2014): sleep schedule regularity, sleep quality, reports of daytime alertness, timing of the main sleep period, patterns of wakefulness preceding or interrupting the main sleep period (sleep efficiency, based on the metrics provided by the under-mattress sensor), and sleep duration. Coaching messages followed three main templates: an insight template which describes what aspect of the participant's sleep needs improving; an education template which provides context on why a sleep issue is important; and a suggestion template that provides a tip on how they can address a given issue (see examples of each in Table 2). Participants were given the messages in a set, where the order of the set was insight, education, and suggestion. Messages were written and saved in a database and included in the IRB-reviewed participant-facing materials. During the intervention phase, message content for a participant on a given day was selected from the database based on automated calculation of metrics for each rule and a system that variably weighted each area, based on the number of activated rules and degree of deviation from what might be considered “optimal” for that rule. The research team assessed the appropriateness of the content selections at their tri-weekly meetings and scheduled relevant SMS based on discussion and consensus. Each set was selected to be the most relevant aspect of sleep for them that particular week, with the next week being potentially a different set.

**Table 2:** Example sleep content.

**Example insight content (base on evidence of**
**significant workday-free day sleep schedule differences)**
Your sleep pattern changes on days off If you change your sleep schedule by an hour or more on days off, going back to work can feel like changing timezones. This is sometimes called “social jetlag.” When you can, it's better to stay consistent across the week. Making small tweaks to your schedule can help you feel more rested in the long run.
**Example educational content**
Make the most of your days off Setting your workday alarm on your days off helps you make the most of the day. But studies also show that a consistent schedule can help you be more productive during the week, too.
**Example suggestion content**
Stay true to your workday schedule If you find it hard to get up on workdays, try getting up at around your typical wake time of XX:XX on your days off. Sleeping late for 2 days can affect your body clock for the other 5.

### Statistical analysis

Given that the main recommendation in the study was for a bedtime/wake-time routine, overall protocol adherence was estimated based on frequency of days in which participants had a wake time (WT) within 30 minutes of the recommended WT. As there was no WT recommendation during the baseline phase, the median WT from the entire baseline phase was used as a surrogate WT target to estimate “adherence” to a regular routine during that phase. Bedtime was not used because the estimate of bedtime provided by the sensor does not accurately reflect the time of getting into bed, but, in most cases, just reflects a time 5 min prior to estimated sleep onset. Along these lines, given that sleep efficiency was calculated from a denominator that does not reflect true time in bed, the normal range for sleep efficiency was set at 90–98% (the 10 and 90th percentile of sleep efficiencies for the entire cohort). Additionally, because of differences in the way sleep is estimated by the sensor relative to the validation analyses performed by Brindle and colleagues (Brindle et al., 2019), the normal range for sleep duration was set at the recommended 7–9 h for a population with our demographics (Hirshkowitz et al., 2015; Watson et al., 2015). The midpoint of the sleep period (MSP) was calculated as the midpoint from sleep onset to final wake time; the normal range for the timing and standard deviation of the MSP were used from the validation study performed by Brindle and colleagues (Brindle et al., 2019). Because daily sleep quality and alertness questions inverted responses, validated thresholds (Brindle et al., 2019) were similarly inverted to define normal vs. not. In addition to examining the absolute and relative number of individual metrics in/out of the normal range on a daily basis, an aggregated daily sleep-health (RU_SATED) (Brindle et al., 2019) score was calculated for each participant by summing the number of sleep health metrics that were in the “normal” range, with a minimum possible score of 0 and maximum of 6.

Categorical variables are presented as percentages, and continuous variables as mean±standard deviation or median and interquartile range for non-Gaussian variables, as confirmed by the Shapiro-Wilk test for normality. Similarly, when normal distributions of variables were evident, period-to-period comparisons of continuous variables were performed using ANOVA (multi-period comparisons), and *post hoc* pairwise comparisons were performed with the *t*-test only in instances where the ANOVA *p*-value was below the significance threshold; otherwise, the Kruskal-Wallis test and *post hoc* Wilcoxon rank sum testing were used. χ^2^ or Fisher's exact test (when counts fell below 5 in any category) was used to compare categorical variables between groups. Pearson coefficients were calculated to demonstrate magnitude of correlation between relevant variables. A statistical threshold of α = 0.05 was set, and, when relevant, Bonferroni correction for multiple comparisons was performed for each major analysis.

Analyses were performed in the R programming language (v4.0.4) (R Core Team, 2019). In order to generate aggregated daily sleep health scores, data were imputed using the mice package for R (van Buuren and Groothuis-Oudshoorn, 2011), assuming that missingness was at random. Otherwise, linear mixed models (LMMs) were fit with the lme4 package (Bates et al., 2015), due to their relative robustness *vis a vis* modest missingness and auto-correlated longitudinal data. The LMMs were used to model general changes of metrics over the course of the study after accounting for various potential sources of confounding: fixed effects for age and sex and random effects (slope and intercept) for study start date and participant (ID). In order to roughly model changes in adherence over time, generalized additive models (GAMs) were used with a penalized cubic regression spline, which had its penalty modified to shrink toward zero at high enough smoothing parameters.

## Results

### Population characteristics

Of the 4,010 potentially eligible individuals who were emailed, 116 completed the screening questionnaire to determine eligibility for study enrollment, based on the inclusion/exclusion criteria (Table 1). Because the sleep schedule recommendation and coaching messages were based on sleep-related behaviors, individuals with sleep schedule irregularity—defined as > 1 h difference in bedtime and/or wake time, at least once a week, on a regular basis—were preferentially recruited. Of those who remained eligible following the screening questionnaire, 37 were consented for participation in the study. Of these, 31 individuals actually participated in the study, with 1 individual (#19) being withdrawn before completion of the study due to their lack of responses to SMS surveys and inability to sleep with the device for > 80% of nights. Four participants' data were excluded from final quantitative analysis due to extrinsic factors that altered their sleep patterns resulting in an inconsistent ability to adhere to the protocol throughout the study period: #4 & #7, due significantly erratic sleep schedules and disruptions potentially due to hardware issues or external factors; #9 and #18, due to extreme changes in sleep duration and schedule due to illness midway through the study. The remaining 26 participants were included in this analysis.

The participants in this study were younger (34.92 ± 10.08 years old) and predominantly male (20M:6F). Despite an effort to recruit volunteers with an irregular sleep schedule, the average standard deviation of the MSP wasn't very high (Tables 3, 4, Baseline column), particularly in relation to the 1:05 min threshold proposed by Brindle et al. (2019). Additionally, in relation to the MIDUS II and MIDUS refresher validation cohorts that Brindle and colleagues used to derive metric cutoffs, this study's participants reported being significantly less alert and had generally later sleep periods and longer sleep duration, with higher efficiency (all *p*-values less than the Bonferroni-corrected α of 0.008; see Supplementary Table 8). The longer sleep duration and higher efficiency may have been, in part, due to the different sleep tracking methods used in this study.

**Table 3:** Comparison of within-participant-averaged RU_SATED metrics over the 3 study phases (2-week baseline, 4-week intervention, 8-week monitoring).

**Metric**	**Baseline mean±SD or median [IQR]**	**Intervention mean±SD or median [IQR]**	**Monitoring mean±SD or median [IQR]**	**Test statistic, *p*-value**	***Post-hoc***
**R**o**U**tine	0:33 [0:23, 1:02]	0:41 [0:27, 0:54]	0:42 [0:29, 0:55]	0.74, 0.48	
**S**leep quality	3.57 [3.15, 3.86]	3.62 [3.31, 4.02]	3.69 [3.34, 3.97]	0.31, 0.74	
**A**lertness	3.54 [3.22, 3.82]	3.68 [3.19, 4.13]	3.73 [3.17, 3.88]	0.23, 0.79	
**T**iming	4:02 [3:27, 5:52]	3:51 [3:13, 5:35]	4:16 [3:44, 5:28]	0.22, 0.80	
**E**fficiency	94.6% [93.3%, 96.0%]	95.5% [93.6%, 95.9%]	95.0% [94.5%, 95.8%]	0.21, 0.81	
**D**uration	7.49 [6.99, 8.03]	7.50 [7.20, 7.78]	7.42 [7.01, 7.79]	0.12, 0.88	
RU_SATED	3.61 [3.30, 4.13]	4.16 [3.18, 4.39]	3.89 [3.39, 4.17]	0.89, 0.41	

For a general sense of this repeated-measures data, each metric was averaged for each participant for each time period and then summary statistics were calculated over the entire cohort. For participant-level data for each metric, see Supplementary Tables 1–7. Normally distributed variables are expressed as mean±SD and analyzed with ANOVA (and *post hoc* t-tests, where appropriate); non-Gaussian variables are expressed as median [IQR] and analyzed with a Kruskal-Wallis test (and *post hoc* Wilcoxon rank sum test, where appropriate). *Post hoc* pairwise comparisons are only provided when the preceding period-level comparison was statistically significant.

**Table 4:** Aggregate of percentage of time spent in the ideal range, by each participant, for each of the RU_SATED metrics over the 3 study phases (2-week baseline, 4-week intervention, 8-week monitoring).

**Metric**	**Baseline** **mean±SD** **or median [IQR]**	**Intervention** **mean±SD** **or median [IQR]**	**Monitoring** **mean±SD** **or median [IQR]**	**Test statistic,** **p-value**	***Post-hoc*** **(higher=better)**
**R**o**U**tine	100.0% [50.0%, 100.0%]	100.0% [74.1%, 100.0%]	87.5% [73.3%, 100.0%]	0.30, 0.74	
**S**leep quality	52.4%±27.2%	56.9%±24.0%	56.2%±27.3%	0.19, 0.83	
**A**lertness	50.2%±34.0%	58.0%±27.8%	52.7%±31.8%	0.40, 0.68	
**T**iming	21.4% [0.0%, 35.1%]	16.3% [0.0%, 49.1%]	9.2% [1.8%, 25.0%]	0.09, 0.92	
**E**fficiency	96.4% [92.9%, 100.0%]	100.0% [93.6%, 100.0%]	98.1% [95.3%, 100.0%]	0.30, 0.74	
**D**uration	54.8%±21.3%	61.8%±17.4%	57.7%±17.7%	0.90, 0.41	

For a general sense of this repeated-measures data, for each metric, the percentage of the period spent in an ideal range (e.g., sleep duration 7–9 h) was calculated for each participant for each time period and then summary statistics were calculated over the entire cohort. Normally distributed variables are expressed as mean±SD and analyzed with ANOVA (and *post hoc* t-tests, where appropriate); non-Gaussian variables are expressed as median [IQR] and analyzed with a Kruskal-Wallis test (and *post hoc* Wilcoxon rank sum test, where appropriate). *Post hoc* pairwise comparisons are only provided when the preceding period-level comparison was statistically significant.

### Adherence

**Figure 2:**
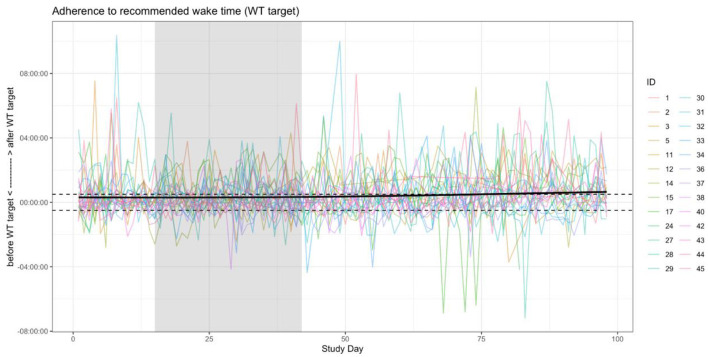
Wake time (WT) adherence. The differences between actual WT and recommended WT are plotted per individual over the course of the study, with positive values indicating an actual WT after the recommended WT. A generalized additive model (GAM) of the form WT difference ~ study day was used to estimate any trend in non-adherence and the point at which the group generally was no longer adherent to their individual WT recommendations, the latter of which was at approximately the 87th day of the study (about 45 days following the end of the intervention). The shaded region indicates the intervention phase, and the horizontal dashed lines indicate the 30-min bounds around the WT recommendation that was considered “adherence”. *Note that the baseline period of the study had no WT recommendation, so median WT for the entire baseline period was used as the reference*.

**Figure 3:**
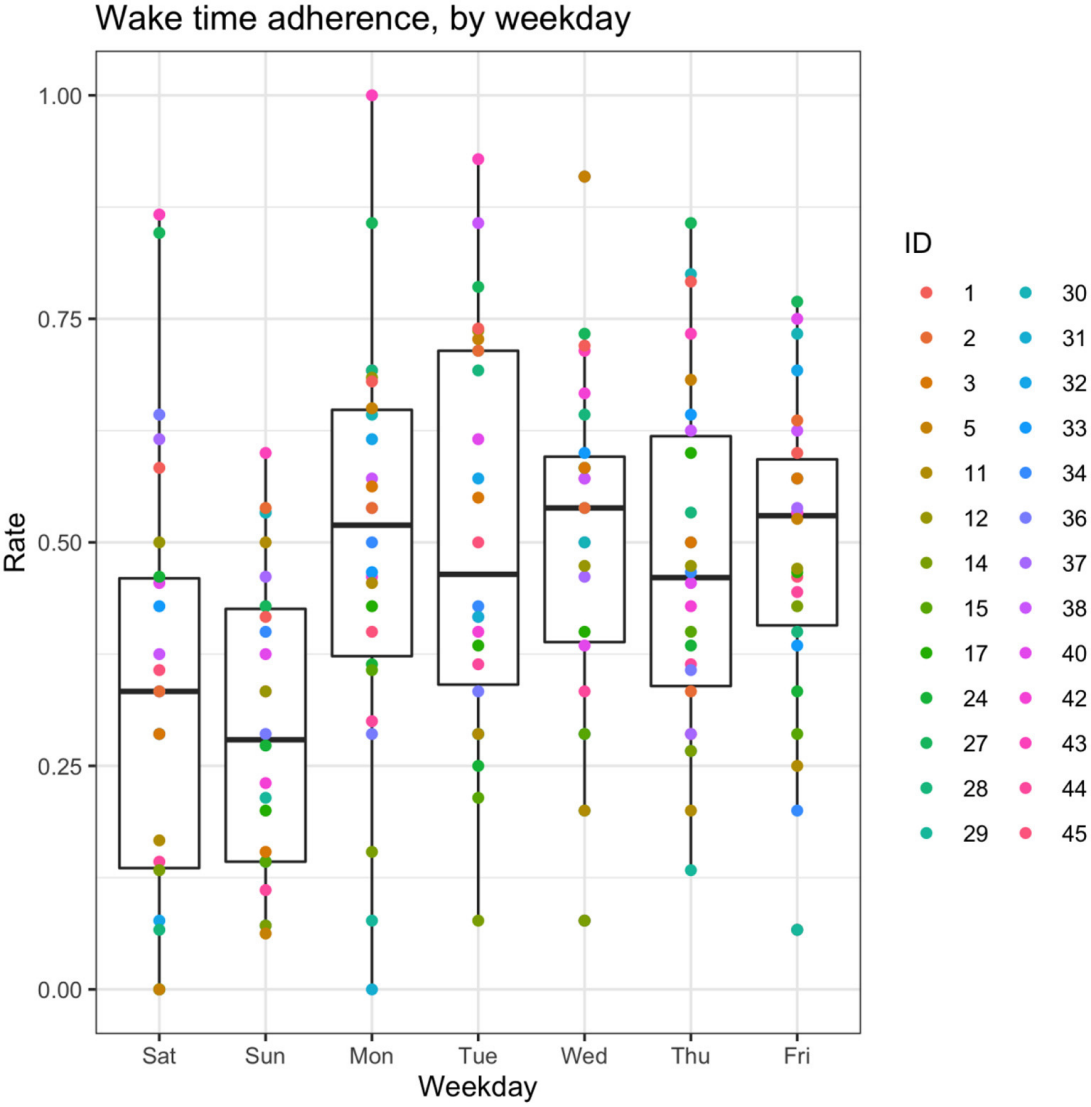
Box-and-whisker plots of adherence rates by weekday. Aggregated wake time (WT) adherence rates, by day of the week. For the box-and-whisker plots: the middle line represents the median, box ends represent 25 and 75th percentiles, and whiskers extend to at most 1.5*IQR beyond the hinge.

Adherence to the recommended wake time (WT) schedule was somewhat inconsistent in this cohort (Figure 2). A shrinkage-penalized, cubic spline, generalized additive model (GAM) was used to ascertain any trend in WT adherence over the course of the study. There appeared to be a tendency toward progressive delay in WT for the cohort as a whole, with participants “falling out” of the recommended WT±30 min window by about the 87th day of the study (45 days after the intervention phase). From another vantage point, looking at weekly rates of adherence (i.e., proportion of the week with actual WT within 30 minutes of the recommended WT), ANOVA suggested that adherence rates differed by study week [*F*_(13, 338)_ 2.05, *p* = 0.017], but, due to a large number of *post hoc* comparisons for the 14 weeks of the study, no pairwise significance was observed. Therefore, linear mixed models were used to model the changes in adherence rates over time, accounting for self-correlation on repeated measures, inter-individual differences, and occasional missing data points. In general, after adjusting for potential confounders (age and sex) as well as start date and inter-individual differences (with random effects), there was a significant main effect for the week of study, which suggested an approximately 1.5% reduction in weekly WT adherence rates over the course of the study (Table 5). As expected, weekend schedule adherence was the lowest and mid-week adherence was the highest [*F*_(6, 175)_ 5.05, *p* = 8.28^*^10^−5^; Tue and Wed > Mon, Thu, Fri > Sat > Sun, by *post hoc* Tukey's honestly significant difference testing] (Figure 3).

**Table 5:** Fixed effects of linear mixed model (LMM) of weekly, wake time (WT)-adherence rates.

	**β**	**SE**	***t*-value**	**df**	***p*-value**
Study Week	−0.015	0.0028	−5.41	1	<0.05
Age	0.0057	0.0034	1.69	1	0.09
Sex (*F*=1)	0.083	0.082	1.02	1	0.31

Fixed effects from the LMM of weekly, WT-adherence rates. The model explained a minor portion of the variance in adherence rates: ${\text{R}}_{\text{marginal}}^{2}$ = 0.09; ${\text{R}}_{\text{conditional}}^{2}$ = 0.39.

Adherence was also explored based on how many consecutive days individuals were able to wake within 30 min of the recommended WT. There was a significant difference in the length of the longest duration of adherence per individual between phases of the study [*F*_(2, 50)_ 3.64, *p* = 0.03], with the intervention phase having longer streaks than the baseline phase on *post hoc* analysis (5.23 ± 3.73 vs. 3.73 ± 1.51 days, respectively; t_(24)_ 2.40, *p* = 0.024) (Supplementary Figure 1). In order to explore the relationship between duration of adherence with subjective reports of alertness and sleep quality, LMMs that accounted for linear and quadratic relationships between length of each participant's longest continuous adherence (“streak”) revealed no statistically significant main effects of adherence length (Supplementary Figures 2, 3).

### Sleep health metrics

Consistent with the study phase comparisons in Tables 3, 4, none of the LMMs separately exploring changes in the 6 RU_SATED metrics demonstrated a main effect for the study day (all *p*-values >0.05), after adjustment for fixed effects of age and sex, and allowing for random slopes and intercepts based on start date and per participant (Table 6). The models' fixed effects explained, at most, a modest 22% of the variance in the metrics, with the only significant effect demonstrating that older age, expectedly, associated with an earlier sleep period (β = 5-min earlier MSP per year, *p* < 0.01). Moreover, the aggregate models' magnitudes of change over the intervention phase—from day 14 to day 42—weren't substantial: 16 min, 19 s increase in the standard deviation of the MSP; 0.18 point improvement in sleep quality perception; 0.30 point improvement in daytime alertness perception; 4 min, 18 s earlier timing of MSP; 0.10% reduction in sleep efficiency; and an 18 min reduction in sleep duration.

**Table 6:** Fixed effects for linear mixed models (LMMs) of changes in RU_SATED metrics over the first 6 weeks of the study.

**Metric**	**Study Day**	**Age**	**Sex (F=1)**	*** ${\text{R}}_{marginal}^{2}$ ***	*** ${\text{R}}_{conditional}^{2}$ ***	**Modeled @d14**	**Modeled @d42**	**Modeled Δ**
**R**o**U**tine	00:00, *p =* 0.57	−00:00, *p =* 0.04	−00:01, *p =* 0.87	0.07	0.44	0:50:25	1:06:44	0:16:19
**S**leep quality	0.01, *p =* 0.11	0.01, *p =* 0.65	0.44, *p =* 0.09	0.03	0.27	3.50	3.68	0.18
**A**lertness	0.01, *p =* 0.06	0.00, *p =* 0.95	0.17, *p =* 0.59	0.02	0.39	3.47	3.76	0.30
**T**iming	−00:00, *p =* 0.47	−00:05, p <0.01	00:30, *p =* 0.51	0.22	0.81	4:30 AM	4:25 AM	−0:04:18
**E**fficiency	0.0%, *p =* 0.85	0.0%, *p =* 0.17	1.5%, *p =* 0.06	0.01	0.13	94.66%	94.56%	−0.10%
**D**uration	0.00, *p =* 0.78	0.00, *p =* 0.80	0.09, *p =* 0.79	0.00	0.20	7.55	7.52	−0.03

The LMMs include adjustments for the following: fixed effects for age and sex and random effects for start date and participant. There were no significant main effects for day of study, after model adjustment, and the models' fixed effects generally explained little of the variance (${\text{R}}_{\text{marginal}}^{2}$ 0.00–0.22) in the metric over the time of the study. For context on the model predictions, an “averaged individual” (34.92 years old and 23.08% female) was used to estimate each metric at the start (day 14) and end (day 42) of the intervention phase, and the approximate magnitude of change over the course of the intervention was expressed as the difference between the end and start of the 4-week intervention phase (Modeled Δ).

**Figure 4:**
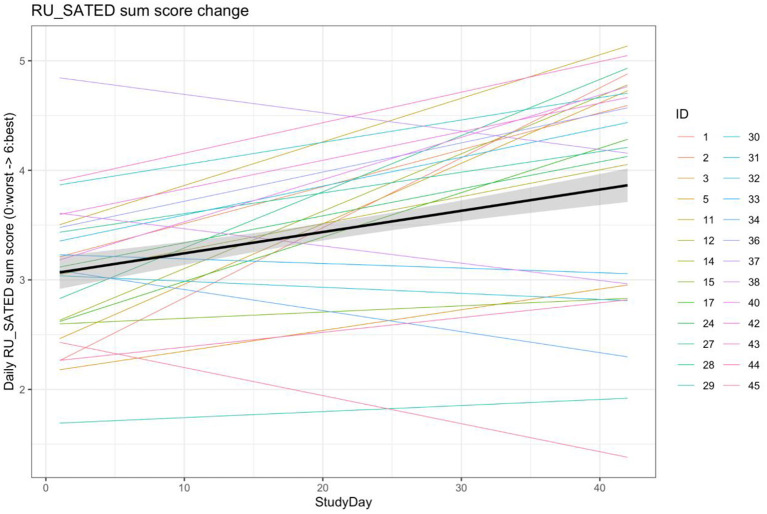
Linear mixed model of RU_SATED score change over the first 6 weeks of the study. Aggregate model and individual models of changes in the RU_SATED sum score over the first 6 weeks-2 baseline and 4 intervention—of the study. RU_SATED scores were calculated using imputed data, to address missingness, and models were adjusted for age and sex as fixed effects, as well as allowing for random slopes/intercepts based on start date and participant ID.

Because the sleep patterns of the participants were so diverse, it was felt that the individual metrics were possibly not an adequate reflection of changes in sleep health resulting from the intervention. As such, for the first 6 weeks of the study (2 baseline weeks and 4 intervention weeks), we performed an analysis of changes in a daily RU_SATED score that was calculated from imputed data (to ensure a full score could be calculated), in which 1 point was added for each metric in the prespecified ideal range, for a maximum possible score of 6 points. After adjusting for fixed effects of age and sex, and allowing for random slopes and intercepts based on start date and per participant, there was a significant, but modest daily improvement in the aggregate RU_SATED score over the course of the study (β = 0.02 point improvement per day, *p* = 1.90^*^10^−13^), which translates to a 0.56-point improvement over the 4 weeks of the intervention phase, in general (Figure 4). Of note, the majority−15/28 (58%)—of the participants demonstrated an improvement in their overall sleep health over the course of the intervention.

**Figure 5:**
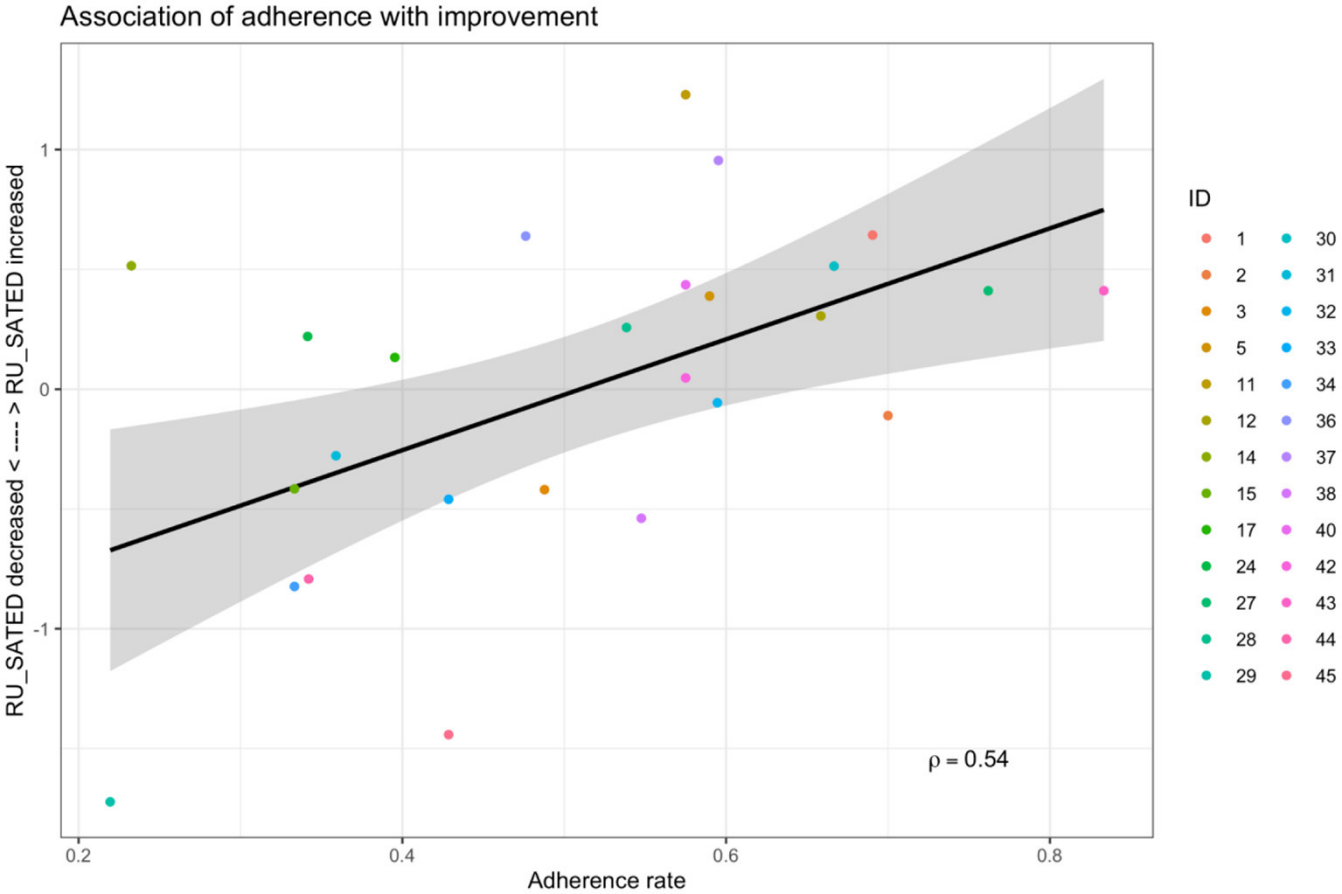
Correlation between wake time-adherence rate and RU_SATED score over the 4-week intervention change.

Given the high degree of variability in both adherence rates and RU_SATED score changes, an analysis was performed to explore whether adherence to the WT recommendation over the 4-week intervention phase correlated with the change in RU_SATED score over this same time period. As demonstrated in Figure 5, there was a moderate positive correlation between adherence rate and RU_SATED score change (ρ = 0.54, *p* = 0.004).

### Qualitative analysis

Upon completion of the intervention phase, participants were given a survey on their experience. Of the 26 responses to the question “To what degree did you feel the insights provided were useful to you?” 8% selected very useful, 72% selected somewhat useful, and 20% selected not at all useful.

## Discussion

Here we present the results of a pilot study of a personalized behavior change intervention focused on improving sleep in the general population, through a 4-week program of sleep schedule (bedtime and wake time) recommendations and SMS-based insights and suggestions. While there were not statistically significant or meaningful changes in the individual sleep metrics derived from the RU_SATED framework, over the 4-week intervention, there was a general trend in improvement of overall sleep health of the cohort, as quantified by an aggregate RU_SATED sum score. This overall improvement over the course of the intervention was evident in the majority of participants, and was significantly associated with adherence to the recommended schedule.

Regarding the improvement in the overall sleep health score, a 0.56-point improvement reflects a >9% improvement in the absolute score—which has a maximum of 6 points. According to the analyses performed by Brindle and colleagues, for each 1-point improvement in the sleep health metric, there is an approximately 10% reduction in cardiometabolic morbidity (OR [95% CI] = 0.901 [0.814–0.997], p = 0.043) (Brindle et al., 2019). As such, if the general improvements realized by the end of the end of the intervention phase were sustained long-term, this would imply a roughly 5% improvement in cardiometabolic morbidity for the cohort, on average, relative to when they started the study.

While efforts were made to prioritize volunteers who could benefit from sleep schedule stabilization, it was evident that there wasn't significant schedule irregularity in the cohort at baseline (Tables 3, 4). Moreover, adherence to a consistent wake time on weekends was lowest (Figure 3), which is concordant with the usual sleep patterns of many individuals (Walch et al., 2016; Åkerstedt et al., 2019). Even though the rates of adherence to the wake time recommendation were quite variable over the 4-week intervention phase (median adherence 50% of days, range 22–86%), it's worth noting that the schedule adherence during this period was generally higher than during the monitoring phase. Reasonable adherence is further supported by the longer consecutive adherence periods during the intervention phase of the study, relative to the baseline phase (5.23 ± 3.73 vs. 3.73 ± 1.51 days, respectively; t_(24)_ 2.40, *p* = 0.024) (Supplementary Figure 1). Additionally, there was an approximately 1.5% reduction in general adherence rates per week over the course of the study, resulting in a progressive delay of participants' general wake times to at least 30-minutes later than the recommendation by about the 45th day following the intervention. As such, it's important to note that the participant experience in the study was meant to resemble a real-world implementation of periodic, data-derived sleep schedule and behavior-change nudges rather than a specific program that is focused on treating a given condition to a therapeutic endpoint. Even in specific digital health interventions—from medication administration to insomnia treatment to weight loss—adherence rates tend to be consistent with those observed in this study (Fenerty et al., 2012; Horsch et al., 2015; Jacobs et al., 2017). Additionally, while causality cannot be inferred from the design of this study, it was observed that higher rates of adherence were correlated with improvement in overall sleep health over the intervention phase (ρ = 0.54, *p* = 0.004). Taken together, these findings suggest that algorithmically generated sleep behavior nudges and schedule recommendations can result in reasonably sustained sleep schedule adherence, which is associated with improvements in a holistic measure of sleep health.

## Limitations

There are a number of limitations to this study, the most notable of which is the fact that there was no control group and the study spanned an artificial change in daily routines related to the implementation of social-distancing and work-from-home policies related to the SARS-Cov2 pandemic (first participant started 2/28/2020, last participant finished 1/1/2021). In order to address potential confounding, models accounted for random effects of study start date. Moreover, these findings may not be generalizable to the general population, given that these individuals were generally younger and disproportionately male, in addition to having sleep patterns that are not reflective of the population at large (Walch et al., 2016; Jaiswal et al., 2020; Jonasdottir et al., 2021; Kocevska et al., 2021). Furthermore, given the convenience sampling method of volunteer participation, there may have been a bias in the population of individuals who participated in this study (e.g., those with an interest in sleep or dissatisfaction with their current sleep patterns). However, these concerns are likely mitigated by the fact that individuals who are inclined to engage with such a sleep, behavior-change program are likely to skew younger and may be searching for ways in which to improve their sleep. Another consideration in interpreting these findings is that the modest sample size may have been inadequately powered relative to our *a priori* estimates, given the wider variation and generally healthier sleep baseline of this population (contributing to smaller effect sizes), particularly in comparison to the cohorts used to derive and validate the RU_SATED metrics (Brindle et al., 2019). Fortunately, the aggregated RU_SATED metric somewhat accounts for individual differences in sleep patterns that may not be directly comparable, and the linear mixed models used also help adjust for between-individual differences. Additionally, by fully automating the algorithmic determination of coaching messages and sleep schedule recommendations (i.e., removing consensus review), sample sizes can greatly increase in future studies. Finally, due to resource limitations and time constraints, the coaching messages were scheduled on a Monday–Wednesday–Friday cadence that may have resulted in messages that lacked salience at the time of receipt. Larger-scale studies of a more automated framework could test various aspects (timing, frequency, etc.) that influence message effectiveness and schedule adherence.

## Future directions

While this study was designed to assess the feasibility of an automated sleep behavior change coaching infrastructure, the involvement of human oversight clearly limited the scalability of such an approach. However, with a fully automated sleep health program, it is possible to reach countless individuals seeking to understand and improve their sleep (potentially making good on the promise of sleep-tracking technologies Chen, 2019). This is particularly important considering the fact that the field of sleep medicine is already struggling to address the abundance of sleep disorders (Thomas et al., 2016; Collen et al., 2020), leaving most individuals without the need for clinical condition management to search on their own for guidance on how to make improvements toward better sleep. As a result individuals may end up mistakenly acting on the preponderance of sleep health misinformation (Robbins et al., 2019) or even developing a sleep disorder as a result of insufficient understanding of how to use their sleep tracking data (Baron et al., 2017). As such, having a robust automated sleep coaching infrastructure could empower the average individual with evidence-based, personalized insights and actions derived from their own sleep data.

## Conclusions

Our findings demonstrate reasonable adherence to and, when more adherent, improvement associated with a sleep wellness coaching framework focused on providing sleep schedule recommendations and coaching insights derived from continuously collected sleep-tracking data in a sample of healthy volunteers sleeping in their natural environment. Importantly, there was a general trend toward overall “better” sleep (as quantified by the total RU_SATED score), with the majority (58%) of the cohort demonstrating improvement despite starting at an already healthy baseline. This suggests that users of such an approach can make improvements to their sleep, based on automated, algorithmic analysis of their longitudinally collected sleep data.

## Data availability statement

The raw data supporting the conclusions of this article will be made available by the authors, without undue reservation.

## Ethics statement

The studies involving human participants were reviewed and approved by Advarra. The patients/participants provided their written informed consent to participate in this study.

## Author contributions

LS, AB, JT, and AJ contributed to conception and design of the study. LS and AJ organized the database. LS, CC, and ZA performed the statistical analysis. LS wrote the first draft of the manuscript. All authors contributed to manuscript revision, read, and approved the submitted version.
