# Supplementary material for: Pilot study of personalized sleep-coaching messages to promote healthy sleeping behaviors
Source: Front Sleep. 2023 Jan 9;1:1071822. doi: 10.3389/frsle.2022.1071822 (PMC12713798; doi:10.3389/frsle.2022.1071822)
Supplement: Supplementary file 1 [file Data_Sheet_1.docx]

**Pilot study of personalized sleep-coaching messages to promote healthy sleeping behaviors**

# Supplemental Materials

| **ID** | **Baseline**  **mean±SD**  **or median [IQR]** | **Intervention**  **mean±SD**  **or median [IQR]** | **Monitoring**  **mean±SD**  **or median [IQR]** | **Test statistic,**  **p-value** | **Post-hoc**  **(lower=better)** |
| --- | --- | --- | --- | --- | --- |
| 1 | 0:30 [0:27, 0:33] | 0:16 [0:10, 0:22] | 0:30 [0:23, 0:33] | 1.69, 0.18 |  |
| 2 | 0:24 [0:15, 0:34] | 0:13 [0:13, 0:24] | 0:23 [0:15, 0:39] | 0.44, 0.64 |  |
| 3 | 1:13 [0:47, 1:39] | 0:36 [0:29, 0:46] | 0:29 [0:21, 0:48] | 0.19, 0.82 |  |
| 5 | 0:47 [0:34, 1:00] | 0:54 [0:51, 0:58] | 0:52 [0:48, 0:54] | 0.22, 0.80 |  |
| 11 | 0:14 [0:13, 0:16] | 0:28 [0:22, 0:32] | 0:47 [0:40, 1:00] | ***4.36, 0.01*** | I<M |
| 12 | 0:24 [0:22, 0:26] | 0:23 [0:22, 0:28] | 0:35 [0:22, 0:48] | 0.47, 0.63 |  |
| 14 | 1:06 [0:54, 1:19] | 0:54 [0:48, 0:58] | 0:48 [0:40, 1:03] | 0.22, 0.80 |  |
| 15 | 0:35 [0:33, 0:36] | 1:07 [1:02, 1:26] | 1:02 [0:47, 1:21] | 2.33, 0.10 |  |
| 17 | 1:09 [1:06, 1:12] | 0:59 [0:57, 1:02] | 1:09 [0:47, 1:58] | 0.42, 0.66 |  |
| 24 | 0:45 [0:45, 0:45] | 0:47 [0:39, 0:54] | 0:29 [0:22, 0:46] | 0.26, 0.77 |  |
| 27 | 0:23 [0:20, 0:25] | 0:17 [0:14, 0:21] | 0:23 [0:21, 0:31] | 1.22, 0.29 |  |
| 28 | 0:46 [0:42, 0:49] | 0:40 [0:30, 0:52] | 0:53 [0:45, 0:57] | 0.70, 0.50 |  |
| 29 | 1:30 [1:28, 1:32] | 1:25 [1:18, 1:33] | 1:29 [1:13, 2:04] | 0.09, 0.91 |  |
| 30 | 0:21 [0:17, 0:25] | 0:19 [0:15, 0:27] | 0:23 [0:17, 0:29] | 0.10, 0.91 |  |
| 31 | 1:25 [1:02, 1:48] | 1:13 [1:00, 1:20] | 0:35 [0:32, 0:53] | 1.47, 0.23 |  |
| 32 | 0:31 [0:27, 0:35] | 0:33 [0:31, 0:38] | 0:19 [0:17, 0:26] | 2.25, 0.10 |  |
| 33 | 0:44 [0:41, 0:47] | 1:01 [0:46, 1:35] | 0:40 [0:34, 0:55] | 1.02, 0.36 |  |
| 34 | 0:25 [0:22, 0:27] | 0:50 [0:42, 0:56] | 0:48 [0:35, 0:56] | 1.25, 0.29 |  |
| 36 | 0:31 [0:27, 0:34] | 0:28 [0:24, 0:38] | 0:34 [0:24, 0:51] | 0.15, 0.86 |  |
| 37 | 0:22 [0:22, 0:22] | 0:35 [0:31, 0:36] | 0:33 [0:25, 0:43] | 1.07, 0.34 |  |
| 38 | 0:20 [0:13, 0:26] | 0:42 [0:28, 1:08] | 1:06 [1:00, 1:13] | 1.33, 0.26 |  |
| 40 | 0:18 [0:17, 0:19] | 0:25 [0:24, 0:33] | 0:27 [0:25, 0:31] | 1.60, 0.20 |  |
| 42 | 0:40 [0:39, 0:41] | 0:28 [0:19, 0:33] | 0:29 [0:19, 0:34] | 1.74, 0.18 |  |
| 43 | 0:20 [0:19, 0:20] | 0:16 [0:13, 0:19] | 0:22 [0:20, 0:26] | 1.40, 0.25 |  |
| 44 | 1:02 [0:57, 1:08] | 0:41 [0:30, 1:03] | 0:59 [0:46, 1:10] | 0.71, 0.49 |  |
| 45 | 1:05 [0:54, 1:17] | 1:19 [1:13, 1:25] | 1:08 [1:04, 1:20] | 0.69, 0.50 |  |

**Supplemental Table 1. Comparison of routine metric, per participant, over the 3 study phases (2-week baseline, 4-week intervention, 8-week monitoring).**

| **ID** | **Baseline**  **mean±SD**  **or median [IQR]** | **Intervention**  **mean±SD**  **or median [IQR]** | **Monitoring**  **mean±SD**  **or median [IQR]** | **Test statistic,**  **p-value** | **Post-hoc**  **(higher=better)** |
| --- | --- | --- | --- | --- | --- |
| 1 | 3.5 [3, 4] | 4 [4, 4] | 4 [3, 4] | 1.70, 0.18 |  |
| 2 | 4 [4, 4.75] | 4 [3, 4] | 4 [3, 4] | 1.24, 0.29 |  |
| 3 | 3 [2.25, 4] | 3 [2.5, 3.5] | 4 [3, 4] | ***3.74, 0.02*** | I<M |
| 5 | 4 [3.75, 5] | 4 [3, 4] | 4 [4, 5] | 1.61, 0.20 |  |
| 11 | 3 [3, 4] | 4 [3, 4] | 4 [3, 4] | 0.39, 0.68 |  |
| 12 | 4 [3, 5] | 4 [3.75, 4.25] | 4 [4, 4] | 0.31, 0.73 |  |
| 14 | 4 [3.25, 4.75] | 4 [3, 5] | 4 [3, 5] | 0.26, 0.77 |  |
| 15 | 4 [3, 4] | 3 [3, 4] | 3 [3, 4] | 1.46, 0.23 |  |
| 17 | 5 [4, 5] | 4 [3, 5] | 4 [3.75, 5] | 2.55, 0.08 |  |
| 24 | 3 [3, 4] | 4 [4, 5] | 4 [3.75, 5] | ***6.09, <0.01*** | B<I,M |
| 27 | 4 [3.25, 4] | 4.5 [4, 5] | 4 [4, 5] | 2.35, 0.10 |  |
| 28 | 3 [2.25, 3.75] | 3 [3, 4] | 3 [3, 4] | 0.22, 0.80 |  |
| 29 | 3 [2, 3.75] | 3 [3, 4] | 3 [2, 3] | ***4.28, 0.01*** | I>M |
| 30 | 4 [3.25, 4] | 4 [4, 5] | 5 [4, 5] | ***4.94, <0.01*** | B<M |
| 31 | 4 [3, 4] | 4 [3, 4] | 4 [3, 4] | 0.17, 0.85 |  |
| 32 | 3 [2, 4] | 3 [2, 4] | 3 [3, 4] | 0.53, 0.59 |  |
| 33 | 4 [3, 5] | 4 [3, 4] | 4 [3, 4] | 0.84, 0.43 |  |
| 34 | 2.5 [2, 3.75] | 2 [2, 3] | 3 [2, 4] | ***5.89, <0.01*** | I<M |
| 36 | 4 [3, 5] | 4 [4, 5] | 4 [3, 4] | 0.70, 0.50 |  |
| 37 | 4 [3.25, 4] | 3.5 [3, 4] | 4 [3, 4] | 1.07, 0.34 |  |
| 38 | 3 [2.25, 4] | 3.5 [3, 4] | 3 [2, 3] | ***3.62, 0.03*** | I>M |
| 40 | 4 [3, 4] | 5 [4, 5] | 4 [4, 5] | ***3.89, 0.02*** | B<I,M |
| 42 | 4 [3, 4] | 5 [4, 5] | 4 [3, 4.75] | 2.71, 0.07 |  |
| 43 | 3 [3, 4] | 4 [3, 5] | 4 [3.75, 5] | ***4.22, 0.01*** | B<I,M |
| 44 | 3 [3, 3.5] | 3 [3, 4] | 3 [3, 4] | 0.16, 0.85 |  |
| 45 | 2.5 [2, 3] | 3 [2, 3] | 3 [2, 3] | 0.88, 0.42 |  |

**Supplemental Table 2. Comparison of sleep quality metric, per participant, over the 3 study phases (2-week baseline, 4-week intervention, 8-week monitoring).** Scores range from 1-5, with higher scores being better.

| **ID** | **Baseline**  **mean±SD**  **or median [IQR]** | **Intervention**  **mean±SD**  **or median [IQR]** | **Monitoring**  **mean±SD**  **or median [IQR]** | **Test statistic,**  **p-value** | **Post-hoc**  **(higher=better)** |
| --- | --- | --- | --- | --- | --- |
| 1 | 3 [3, 4] | 5 [4, 5] | 4 [3, 5] | ***3.98, 0.02*** | B,M<I |
| 2 | 4 [3, 4] | 3 [2.75, 4] | 3 [3, 4] | 1.35, 0.26 |  |
| 3 | 3 [3, 4] | 3 [2, 4] | 4 [3, 5] | ***4.22, 0.01*** | I<M |
| 5 | 4 [3, 5] | 4 [3, 4] | 4 [3, 5] | 1.01, 0.37 |  |
| 11 | 4 [3, 4] | 4 [3, 4] | 4 [3, 4] | 0.66, 0.52 |  |
| 12 | 4 [4, 5] | 4 [3.75, 5] | 4 [3, 5] | 0.20, 0.82 |  |
| 14 | 3.5 [2.25, 4] | 4 [3, 5] | 5 [3, 5] | 1.19, 0.31 |  |
| 15 | 2.5 [1, 3] | 4 [3, 4] | 4 [3, 4] | ***5.96, <0.01*** | B<I,M |
| 17 | 4.5 [4, 5] | 4 [4, 5] | 4 [3, 4] | ***4.15, 0.02*** | B,I>M |
| 24 | 4 [3, 4] | 5 [4, 5] | 5 [4, 5] | ***6.38, <0.01*** | B<I,M |
| 27 | 3 [2.25, 4] | 4 [4, 5] | 4 [4, 4] | ***6.67, <0.01*** | B<I,M |
| 28 | 3 [3, 3.75] | 3 [2, 3] | 3 [3, 3] | 1.67, 0.19 |  |
| 29 | 2.5 [2, 3] | 3 [3, 4] | 3 [2, 3] | ***5.24, <0.01*** | I>M |
| 30 | 4 [3.25, 5] | 4.5 [4, 5] | 5 [4, 5] | ***4.52, 0.01*** | B<M |
| 31 | 4 [3.25, 4] | 4 [3, 4] | 3.5 [3, 4] | 0.75, 0.47 |  |
| 32 | 3 [3, 4] | 3 [3, 4] | 3 [3, 3] | ***3.36, 0.03*** | I>M |
| 33 | 4 [3, 4.75] | 4 [3, 4] | 4 [3, 4] | 0.27, 0.76 |  |
| 34 | 3 [3, 4] | 3 [2, 3] | 3 [3, 4] | ***4.70, <0.01*** | B,M>I |
| 36 | 4.5 [4, 5] | 4 [3, 5] | 4 [3.75, 5] | 0.48, 0.62 |  |
| 37 | 5 [4.25, 5] | 4 [3.75, 4] | 4 [3, 4] | ***12.35, <0.01*** | B>I,M |
| 38 | 3 [3, 3.75] | 3.5 [3, 4] | 3 [2.25, 3] | ***3.46, 0.03*** | I>M |
| 40 | 3 [3, 4] | 4 [4, 5] | 4 [4, 5] | ***4.61, <0.01*** | B<I,M |
| 42 | 3 [3, 4] | 4 [3, 4] | 4 [3, 4] | 2.29, 0.10 |  |
| 43 | 4 [4, 5] | 4 [4, 5] | 5 [4, 5] | 0.30, 0.74 |  |
| 44 | 3 [3, 4] | 3 [3, 4] | 3 [3, 4] | 0.47, 0.63 |  |
| 45 | 2.5 [2, 3] | 3 [2, 3] | 3 [2, 3] | 0.76, 0.47 |  |

**Supplemental Table 3. Comparison of alertness metric, per participant, over the 3 study phases (2-week baseline, 4-week intervention, 8-week monitoring).** Scores range from 1-5, with higher scores being better.

| **ID** | **Baseline**  **mean±SD**  **or median [IQR]** | **Intervention**  **mean±SD**  **or median [IQR]** | **Monitoring**  **mean±SD**  **or median [IQR]** | **Test statistic,**  **p-value** | **Post-hoc** |
| --- | --- | --- | --- | --- | --- |
| 1 | 3:50 [3:25, 4:04] | 3:50 [3:36, 3:50] | 3:50 [3:36, 4:08] | 1.80, 0.17 |  |
| 2 | 2:09 [2:09, 2:24] | 2:24 [2:24, 2:52] | 2:38 [2:24, 2:52] | ***5.45, <0.01*** | B<I,M |
| 3 | 4:33 [4:04, 4:48] | 5:02 [4:48, 5:16] | 5:02 [4:48, 5:16] | 2.76, 0.06 |  |
| 5 | 3:43 [3:18, 4:08] | 3:36 [3:14, 4:19] | 3:50 [3:36, 4:19] | 0.64, 0.53 |  |
| 11 | 2:52 [2:52, 3:07] | 2:52 [2:38, 3:18] | 4:19 [3:50, 4:48] | ***23.43, <0.01*** | B,I<M |
| 12 | 6:04±0:24 | 4:59±0:30 | 5:09±0:42 | ***14.47, <0.01*** | B>I,M |
| 14 | 4:04 [3:25, 4:33] | 3:36 [3:07, 4:33] | 4:33 [4:04, 5:02] | ***8.50, <0.01*** | I<M |
| 15 | 6:02±0:34 | 7:07±1:27 | 7:03±1:12 | ***4.42, 0.01*** | B<I,M |
| 17 | 5:24 [5:02, 6:43] | 5:31 [5:02, 6:21] | 5:38 [5:02, 6:14] | 0.04, 0.97 |  |
| 24 | 4:04 [3:14, 4:19] | 4:04 [3:50, 4:33] | 3:36 [3:21, 4:08] | ***3.10, 0.05*** | I>M |
| 27 | 1:40 [1:26, 1:51] | 1:26 [1:22, 1:44] | 1:26 [1:12, 1:40] | 0.42, 0.65 |  |
| 28 | 3:14 [2:52, 3:36] | 2:38 [2:24, 3:07] | 2:52 [2:24, 3:21] | 2.98, 0.05 |  |
| 29 | 8:09 [7:01, 8:38] | 8:24 [7:48, 9:07] | 9:21 [8:06, 10:19] | ***3.87, 0.02*** | B,I<M |
| 30 | 5:45 [5:31, 6:00] | 5:31 [5:27, 6:00] | 5:31 [5:02, 5:45] | ***5.14, <0.01*** | B,I>M |
| 31 | 6:43 [6:00, 7:12] | 6:00 [5:16, 6:28] | 5:31 [5:02, 5:45] | ***10.23, <0.01*** | B,I>M |
| 32 | 2:31 [2:09, 2:49] | 2:24 [1:55, 2:38] | 2:38 [2:24, 2:52] | 2.91, 0.05 |  |
| 33 | 7:40 [7:04, 7:55] | 7:12 [6:57, 7:40] | 7:12 [6:28, 7:40] | 1.49, 0.23 |  |
| 34 | 3:43±0:25 | 3:15±0:51 | 3:43±0:48 | ***3.65, 0.03*** | B,M>I |
| 36 | 6:50 [6:43, 7:48] | 6:57 [6:28, 7:33] | 6:43 [6:28, 7:01] | 1.83, 0.16 |  |
| 37 | 3:12±0:22 | 2:42±0:35 | 3:12±0:56 | ***4.14, 0.02*** | B,M>I |
| 38 | 2:31 [2:13, 2:38] | 3:07 [2:34, 3:39] | 3:50 [3:21, 4:37] | ***6.86, <0.01*** | B<I,M |
| 40 | 4:33 [4:19, 4:48] | 4:19 [4:04, 4:48] | 4:48 [4:19, 5:16] | ***3.19, 0.04*** | I<M |
| 42 | 3:39±0:40 | 3:38±0:27 | 4:02±0:31 | ***6.38, <0.01*** | I<M |
| 43 | 3:57 [3:36, 4:04] | 3:50 [3:36, 3:50] | 3:36 [3:21, 3:54] | 0.66, 0.51 |  |
| 44 | 5:45 [5:16, 6:07] | 5:16 [4:55, 5:52] | 6:28 [5:42, 7:12] | ***7.67, <0.01*** | I<M |
| 45 | 4:04 [3:36, 4:19] | 3:50 [3:21, 4:19] | 3:50 [3:36, 4:44] | 0.38, 0.68 |  |

**Supplemental Table 4. Comparison of timing metric, per participant, over the 3 study phases (2-week baseline, 4-week intervention, 8-week monitoring).** Time of day expressed in 24-hour notation.

| **ID** | **Baseline**  **mean±SD**  **or median [IQR]** | **Intervention**  **mean±SD**  **or median [IQR]** | **Monitoring**  **mean±SD**  **or median [IQR]** | **Test statistic,**  **p-value** | **Post-hoc** |
| --- | --- | --- | --- | --- | --- |
| 1 | 97.0% [95.3%, 97.0%] | 97.0% [96.0%, 97.0%] | 97.0% [95.0%, 97.0%] | 0.35, 0.71 |  |
| 2 | 96.0% [95.0%, 96.8%] | 96.5% [95.0%, 97.0%] | 96.0% [95.0%, 97.0%] | 0.54, 0.58 |  |
| 3 | 96.0% [91.3%, 97.8%] | 97.0% [95.5%, 97.0%] | 96.0% [95.0%, 97.0%] | 0.98, 0.38 |  |
| 5 | 97.0% [97.0%, 97.0%] | 97.0% [96.5%, 97.0%] | 97.0% [96.0%, 97.0%] | 0.89, 0.41 |  |
| 11 | 95.0% [93.0%, 97.0%] | 96.0% [95.0%, 97.0%] | 96.0% [94.0%, 97.5%] | 0.93, 0.39 |  |
| 12 | 96.0% [95.0%, 96.0%] | 96.0% [95.0%, 97.0%] | 96.0% [94.8%, 97.0%] | 0.07, 0.93 |  |
| 14 | 95.5% [91.8%, 98.0%] | 97.0% [97.0%, 97.0%] | 97.0% [96.0%, 97.0%] | 0.80, 0.45 |  |
| 15 | 97.0% [95.3%, 97.0%] | 96.5% [92.8%, 97.0%] | 97.0% [95.0%, 97.0%] | 0.35, 0.70 |  |
| 17 | 98.0% [97.0%, 98.0%] | 98.0% [97.5%, 98.0%] | 98.0% [97.0%, 98.0%] | 1.81, 0.16 |  |
| 24 | 95.5% [93.0%, 97.0%] | 96.0% [94.0%, 97.0%] | 97.0% [96.0%, 97.0%] | 1.41, 0.24 |  |
| 27 | 97.0% [97.0%, 97.0%] | 97.0% [97.0%, 97.0%] | 97.0% [97.0%, 97.0%] | 0.03, 0.97 |  |
| 28 | 97.0% [97.0%, 97.0%] | 95.0% [91.0%, 97.0%] | 95.5% [91.0%, 97.0%] | 2.79, 0.06 |  |
| 29 | 95.5% [93.5%, 97.0%] | 96.0% [96.0%, 97.0%] | 96.0% [94.0%, 97.0%] | 1.28, 0.28 |  |
| 30 | 95.0% [88.8%, 96.8%] | 95.0% [93.0%, 97.0%] | 95.0% [93.0%, 97.0%] | 0.23, 0.79 |  |
| 31 | 97.0% [90.0%, 97.8%] | 96.0% [94.0%, 97.0%] | 95.5% [88.3%, 97.0%] | 0.92, 0.40 |  |
| 32 | 95.0% [94.0%, 95.8%] | 95.0% [93.0%, 96.0%] | 96.0% [95.0%, 97.0%] | ***4.23, 0.01*** | B,I<M |
| 33 | 97.0% [96.3%, 97.0%] | 97.0% [96.0%, 97.3%] | 97.0% [97.0%, 98.0%] | 0.49, 0.61 |  |
| 34 | 96.5% [92.8%, 97.0%] | 95.0% [92.0%, 97.0%] | 96.0% [95.0%, 97.0%] | 1.60, 0.20 |  |
| 36 | 97.0% [97.0%, 97.8%] | 97.0% [97.0%, 98.0%] | 97.0% [97.0%, 97.0%] | 0.47, 0.63 |  |
| 37 | 96.0% [93.0%, 97.0%] | 94.5% [90.8%, 97.0%] | 97.0% [94.0%, 97.0%] | 1.99, 0.14 |  |
| 38 | 97.0% [96.3%, 97.0%] | 97.0% [94.8%, 97.0%] | 96.0% [95.3%, 97.0%] | 1.85, 0.16 |  |
| 40 | 96.0% [95.0%, 97.0%] | 97.0% [95.5%, 97.0%] | 97.0% [94.0%, 97.0%] | 0.83, 0.44 |  |
| 42 | 96.5% [96.0%, 97.0%] | 97.0% [97.0%, 97.0%] | 97.0% [96.0%, 97.0%] | 1.52, 0.22 |  |
| 43 | 97.0% [95.3%, 97.0%] | 97.0% [97.0%, 97.3%] | 97.0% [97.0%, 97.0%] | ***3.53, 0.03*** | B<I,M |
| 44 | 96.0% [91.5%, 97.0%] | 90.0% [84.5%, 94.5%] | 86.5% [79.5%, 89.3%] | ***7.42, <0.01*** | B>I>M |
| 45 | 93.0% [87.5%, 96.8%] | 95.0% [88.8%, 97.0%] | 90.0% [83.3%, 93.0%] | 2.25, 0.11 |  |

**Supplemental Table 5. Comparison of efficiency metric, per participant, over the 3 study phases (2-week baseline, 4-week intervention, 8-week monitoring).**

| **ID** | **Baseline**  **mean±SD**  **or median [IQR]** | **Intervention**  **mean±SD**  **or median [IQR]** | **Monitoring**  **mean±SD**  **or median [IQR]** | **Test statistic,**  **p-value** | **Post-hoc** |
| --- | --- | --- | --- | --- | --- |
| 1 | 6.64 [6.44, 7.08] | 7.08 [6.61, 7.39] | 7.16 [6.50, 7.44] | 0.83, 0.43 |  |
| 2 | 8.05 [7.86, 8.19] | 8.24 [8.06, 9.36] | 8.75 [8.08, 9.22] | ***3.02, 0.05*** | B<M |
| 3 | 8.11 [6.96, 8.98] | 8.10 [7.66, 8.72] | 8.20 [7.67, 8.78] | 0.01, 0.99 |  |
| 5 | 7.98 [7.34, 8.39] | 7.57 [7.01, 8.14] | 7.40 [7.02, 7.85] | 1.99, 0.14 |  |
| 11 | 7.49 [7.04, 7.81] | 7.68 [7.28, 8.13] | 7.68 [7.35, 8.48] | 0.73, 0.48 |  |
| 12 | 7.23 [7.17, 7.90] | 7.35 [6.90, 7.67] | 7.31 [6.97, 7.62] | 0.20, 0.82 |  |
| 14 | 8.69 [7.97, 9.26] | 7.75 [7.08, 8.07] | 7.72 [7.27, 8.30] | ***3.03, 0.05*** | B>I,M |
| 15 | 7.33±1.27 | 7.21±1.69 | 6.91±1.50 | 0.61, 0.55 |  |
| 17 | 9.00 [8.46, 9.33] | 8.65 [8.28, 8.92] | 8.61 [8.10, 9.52] | 0.48, 0.62 |  |
| 24 | 6.69 [6.51, 7.23] | 7.67 [7.17, 8.19] | 6.84 [6.25, 7.61] | ***3.05, 0.05*** | I>M |
| 27 | 7.05 [6.27, 7.52] | 7.17 [6.90, 7.42] | 6.93 [6.78, 7.38] | 0.94, 0.39 |  |
| 28 | 7.00 [6.57, 7.15] | 7.48 [7.15, 7.93] | 7.50 [6.92, 8.26] | 2.74, 0.06 |  |
| 29 | 6.82±2.10 | 6.43±1.56 | 6.69±1.77 | 0.28, 0.75 |  |
| 30 | 7.44 [6.78, 7.88] | 7.49 [6.97, 7.89] | 7.57 [7.27, 7.98] | 1.23, 0.29 |  |
| 31 | 8.05 [7.07, 9.02] | 8.05 [7.38, 8.85] | 7.25 [6.58, 7.77] | ***7.19, <0.01*** | B,I>M |
| 32 | 8.05 [7.87, 8.65] | 7.95 [7.13, 8.48] | 8.08 [7.75, 8.50] | 0.87, 0.42 |  |
| 33 | 6.77 [6.38, 9.01] | 7.21 [6.12, 8.48] | 7.63 [5.70, 8.82] | 0.04, 0.96 |  |
| 34 | 6.81 [6.02, 7.27] | 7.00 [6.03, 7.76] | 7.63 [6.60, 8.15] | 2.04, 0.13 |  |
| 36 | 7.67 [6.89, 8.31] | 7.53 [7.00, 8.47] | 7.43 [6.98, 8.14] | 0.04, 0.96 |  |
| 37 | 7.85 [7.42, 8.12] | 7.45 [7.07, 8.17] | 7.38 [6.88, 7.97] | 1.39, 0.25 |  |
| 38 | 6.92 [6.84, 7.65] | 6.76 [5.87, 7.19] | 5.93 [4.71, 6.92] | ***3.12, 0.04*** | B>I,M |
| 40 | 6.99±0.97 | 7.20±0.56 | 7.31±0.75 | 0.96, 0.39 |  |
| 42 | 6.44±1.16 | 7.15±0.76 | 6.99±1.05 | 2.19, 0.12 |  |
| 43 | 7.81 [7.56, 8.01] | 7.81 [7.61, 8.15] | 7.82 [7.46, 8.14] | 0.08, 0.92 |  |
| 44 | 6.93 [6.41, 7.35] | 7.10 [6.70, 7.71] | 6.92 [6.48, 7.50] | 0.59, 0.55 |  |
| 45 | 8.10 [7.51, 8.96] | 8.45 [7.55, 9.53] | 8.59 [7.87, 9.85] | 0.65, 0.52 |  |

**Supplemental Table 6. Comparison of duration metric, per participant, over the 3 study phases (2-week baseline, 4-week intervention, 8-week monitoring).**

| **ID** | **Baseline**  **mean±SD or median [IQR]** | **Intervention**  **mean±SD or median [IQR]** | **Monitoring**  **mean±SD or median [IQR]** | **Test statistic,**  **p-value** | **Post-hoc**  **(higher=better)** |
| --- | --- | --- | --- | --- | --- |
| 1 | 3.5 [3, 4] | 5 [4, 5] | 4 [3, 5] | 2.52, 0.08 |  |
| 2 | 5 [4, 5] | 4.5 [4, 5] | 5 [4, 5] | 0.04, 0.96 |  |
| 3 | 3 [1.25, 3.75] | 3 [2, 4] | 4 [3, 5] | ***5.76, <0.01*** | B,I<M |
| 5 | 4 [3, 5] | 4 [3.5, 5] | 4 [4, 5] | 0.48, 0.62 |  |
| 11 | 4.5 [4, 5.75] | 5 [4.25, 6] | 4 [4, 5] | ***3.56, 0.03*** | I>M |
| 12 | 4 [4, 5] | 4 [4, 5] | 4 [4, 5] | 0.00, 1.00 |  |
| 14 | 3.5 [2.25, 4.75] | 4 [4, 5] | 4 [3, 5] | 1.84, 0.16 |  |
| 15 | 3 [3, 4] | 3 [2, 4] | 3 [2, 4] | 0.71, 0.49 |  |
| 17 | 4 [3, 4] | 4 [3, 4.5] | 4 [2.75, 4] | 1.14, 0.32 |  |
| 24 | 3.5 [3, 4] | 5 [4, 5] | 4 [3.75, 5] | 2.97, 0.05 |  |
| 27 | 3.5 [3, 4] | 4 [4, 5] | 4 [4, 5] | ***3.19, 0.04*** | B<I,M |
| 28 | 4 [3, 4] | 4 [3, 4] | 4 [3, 4] | 2.03, 0.13 |  |
| 29 | 2 [1, 2.75] | 2 [1, 3] | 2 [1, 2] | 0.08, 0.92 |  |
| 30 | 4 [4, 5] | 4.5 [4, 5] | 5 [4, 5] | 2.41, 0.09 |  |
| 31 | 3 [2.25, 4] | 3 [2, 4] | 3 [2, 4] | 0.23, 0.80 |  |
| 32 | 4 [3.25, 5] | 4 [3, 5] | 4 [4, 4] | 0.20, 0.82 |  |
| 33 | 4 [3, 4] | 3 [2, 4.25] | 4 [3, 5] | 0.50, 0.60 |  |
| 34 | 3 [2, 4] | 3 [2, 3] | 4 [3, 4] | ***4.18, 0.02*** | I<M |
| 36 | 4 [4, 5] | 4 [4, 5] | 4 [4, 5] | 0.09, 0.92 |  |
| 37 | 5 [5, 6] | 5 [4, 5.25] | 4 [3, 5] | ***4.04, 0.02*** | B>M |
| 38 | 4 [3, 4.75] | 4 [3, 4] | 2 [2, 3] | ***5.45, <0.01*** | B,I>M |
| 40 | 4 [3, 4] | 5 [4, 5] | 4 [4, 5] | ***3.76, 0.02*** | B<I,M |
| 42 | 4 [2.75, 5] | 4.5 [4, 5] | 4 [3, 5] | 2.05, 0.13 |  |
| 43 | 4 [4, 5] | 5 [4, 5] | 5 [4, 5] | 1.28, 0.28 |  |
| 44 | 2 [1.5, 3] | 3 [2, 3] | 2 [1, 3] | 2.20, 0.11 |  |
| 45 | 2.5 [2, 3] | 2 [1, 2] | 2 [1, 2] | 2.22, 0.11 |  |

**Supplemental Table 7. Comparison of composite RU_SATED sum score, per participant, over the 3 study phases (2-week baseline, 4-week intervention, 8-week monitoring).**

|  | **SBC vs MIDUS II** | ***t*-stat, *p*-value** | **SBC vs MIDUS refresher** | ***t*-stat, *p*-value** |
| --- | --- | --- | --- | --- |
| RoUtine | -0:09:23 | -1.88, 0.07 | -0:10:26 | -2.14, 0.04 |
| Sleep quality | -0.05 | -0.49, 0.63 | -0.09 | -0.85, 0.40 |
| Alertness | -0.43 | **-3.87, <0.008** | -0.41 | **-3.59, <0.008** |
| Timing | 1:21:02 | **3.88, <0.008** | 1:21:42 | **3.95, <0.008** |
| Efficiency | 15.2% | **24.70, <0.008** | 15.0% | **19.67, <0.008** |
| Duration | 83.51 | **9.92, <0.008** | 81.48 | **9.24, <0.008** |

**Supplemental Table 8. Comparison of aggregate metrics between this cohort at baseline and the summary statistics from the MIDUS II and MIDUS refresher cohorts used in the Brindle *et al.* RU_SATED validation.** For each of the sleep health dimensions, differences in the means are expressed along with *t*-statistics and *p*-values (relative to the Bonferroni-adjusted α threshold of 0.008, accounting for 6 comparisons) in order to estimate statistical significance of the differences of this cohort from the derivation cohorts.


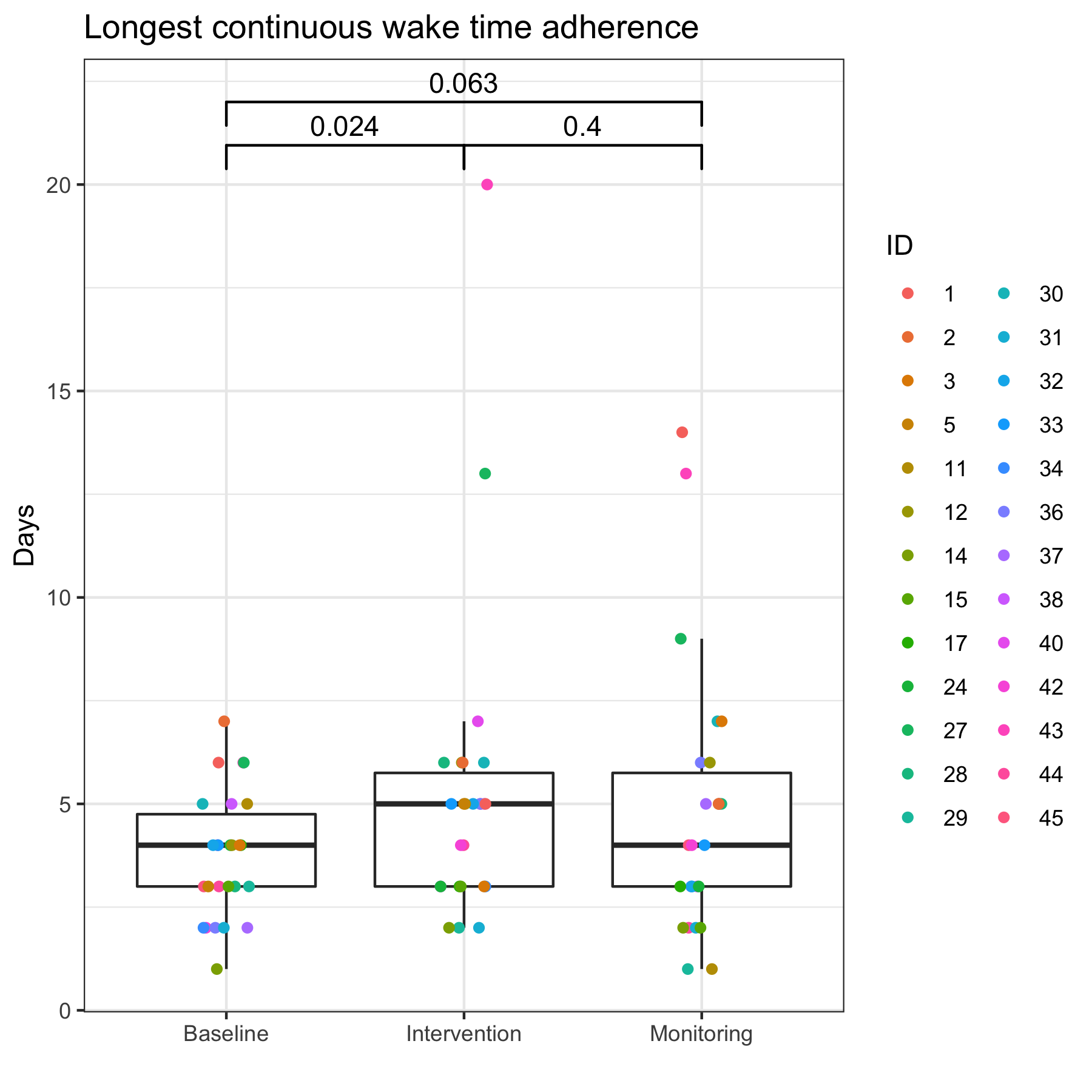


**Supplemental Figure 1. Comparison of the longest number of consecutive days adhering to the wake time (WT) recommendation.** Adherence was calculated per period and per individual. A repeated measures ANOVA was performed, indicating a significant effect of period (F(2,50) 3.64, p 0.03). *Post hoc* *t*-test p-values are provided in the figure for each pairwise comparison.


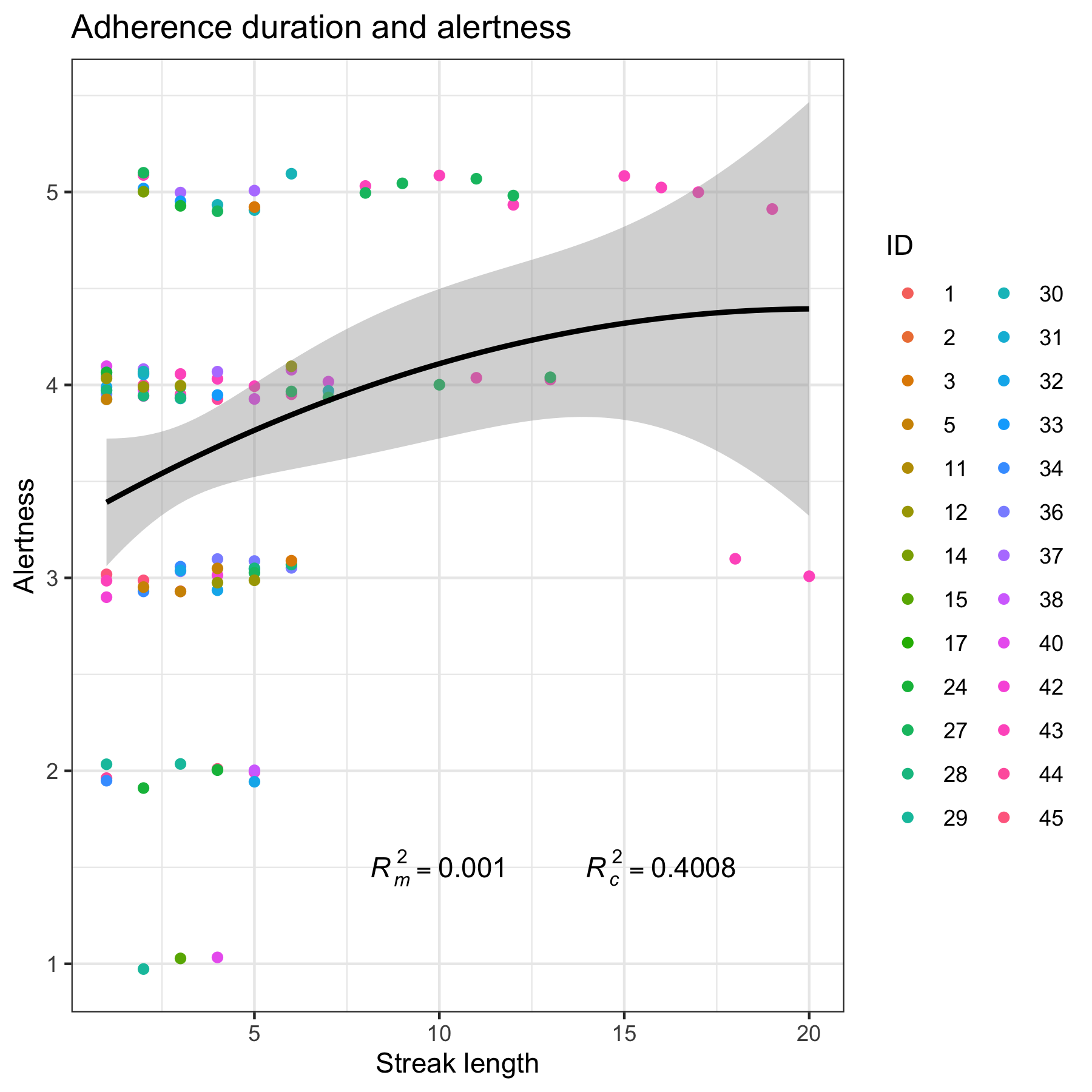


**Supplemental Figure 2. Association of alertness reports with longest duration of adherence per individual.** The linear mixed model (LMM) accounting for participant-level random effects, demonstrated no significant main effects for linear or quadratic terms in the model. Abbreviations: *R^2^_m_*=marginal *R^2^*; *R^2^_c_*=conditional *R^2^* *Note: slight vertical jitter was introduced into data points for visualization purposes.*


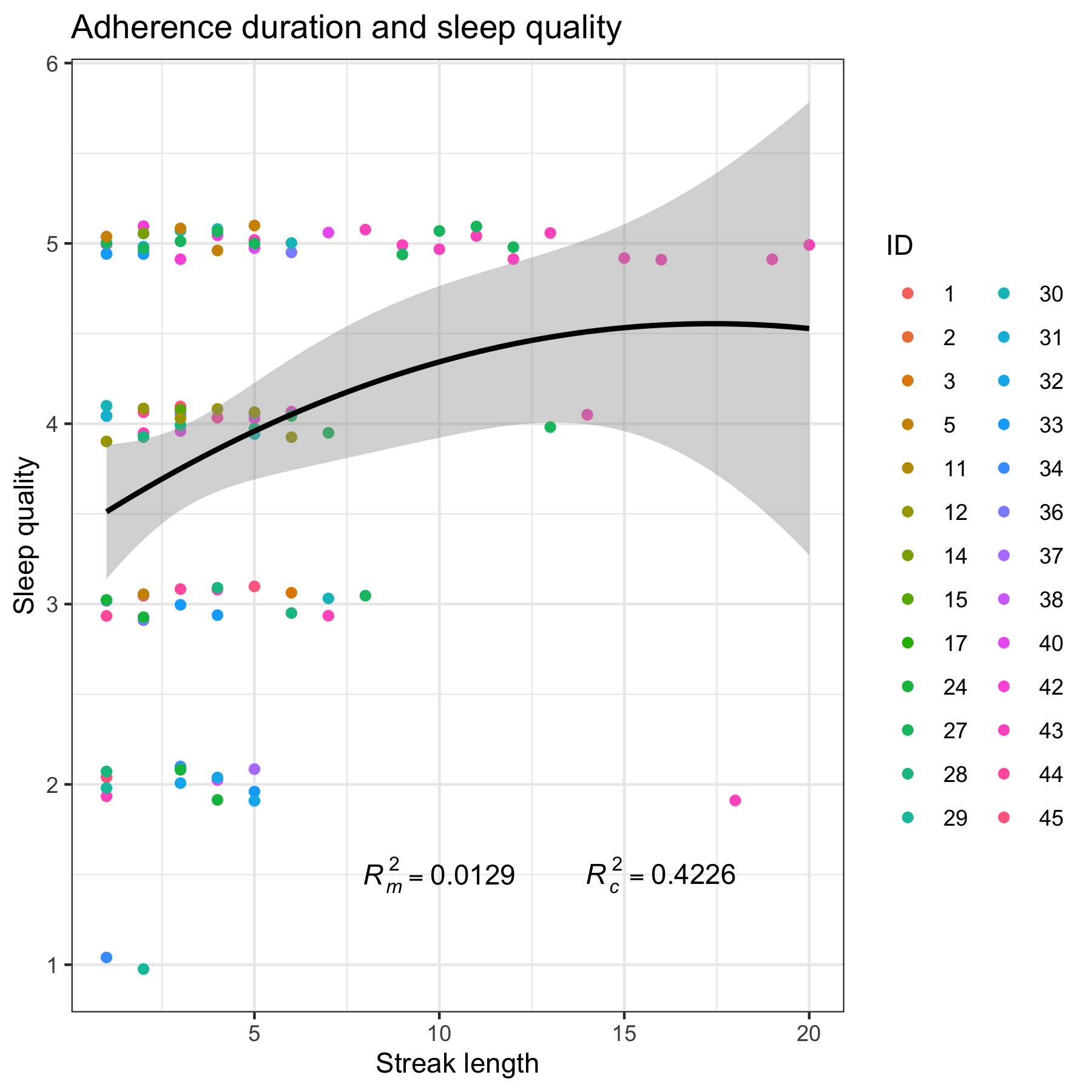


**Supplemental Figure 3. Association of sleep quality reports with longest duration of adherence per individual.** The linear mixed model (LMM) accounting for participant-level random effects, demonstrated no significant main effects for linear or quadratic terms in the model. Abbreviations: *R^2^_m_*=marginal *R^2^*; *R^2^_c_*=conditional *R^2^* *Note: slight vertical jitter was introduced into data points for visualization purposes.*
